# Five key aspects of metaproteomics as a tool to understand functional interactions in host-associated microbiomes

**DOI:** 10.1371/journal.ppat.1009245

**Published:** 2021-02-25

**Authors:** Fernanda Salvato, Robert L. Hettich, Manuel Kleiner

**Affiliations:** 1 Department of Plant and Microbial Biology, North Carolina State University, Raleigh, North Carolina, United States of America; 2 Oak Ridge National Laboratory, Biosciences Division, Oak Ridge, Tennessee, United States of America; Geisel School of Medicine at Dartmouth, UNITED STATES

Host-associated microbial communities (microbiomes) play critical roles in human, animal, and plant health and development. However, interactions between the host, members of the microbiome, and invading pathogens are in most cases still poorly understood. Such interactions are multidimensional [1] and can alter the taxonomic composition and/or the functional metabolic activities of the microbiome in response to disease or treatment conditions. For example, after 2 days of antibiotic treatment, the mouse gut microbiome is altered and more susceptible to invasion by the pathogen *Clostridioides difficile* [2]. Studies of these multidimensional interactions have been fueled by the ability to use high-throughput sequencing of phylogenetic marker genes to profile microbial community composition and shotgun metagenomics to profile functional potential [3]. However, many protein-coding genes predicted from metagenomes are not necessarily expressed under a given condition, and thus, it is difficult to assess the activities and functional interactions in microbial communities based on DNA sequencing data alone [4]. The physiological and pathological processes expressed in these communities under specific conditions are better reflected by the abundances of transcripts or proteins [5,6]. In this Pearl, we provide a brief introduction to metaproteomics, which is a tool for the large-scale analysis of proteins in microbiomes that allows researchers to address a diversity of questions related to functions and interactions in microbiomes [7]. The term “metaproteomics” was first used in 2004 for “the large-scale characterization of the entire protein complement of environmental microbiota at a given point in time” [8], and since then, a large array of metaproteomics approaches have been developed [7]. Our objective in this Pearl is to highlight what we feel are 5 essential elements to be considered for a metaproteomics research campaign and to introduce nonexpert readers to the topic without going into too much technical detail.

## What information can be gained using metaproteomics?

Basic metaproteomes can provide diverse types of information about microbiomes, including composition, abundance dynamics, and the metabolism and physiology of individual members, including the host. Additionally, gene expression responses to changing conditions, localization of potential host–microbe interaction proteins within a host–microbe system, and the uptake of substrates labeled with heavy carbon or nitrogen can be determined [7].

Below we provide selected examples of specific metaproteomics applications in the area of microbiome research to illustrate the diversity of questions that can be addressed with metaproteomics. These examples are not exhaustive, and we emphasize that metaproteomics has been, and will continue to be, used to answer a wide range of questions for a variety of biological research arenas.

Metaproteomics can measure microbial gene expression and help identify genes underlying a phenotype. For example, by analyzing the metaproteome of a uropathogenic biofilm grown in a bladder catheter, Lassek and colleagues [9] were able to identify the bacterial community structure and specific functional roles of these microbes. They found that catheters were dominated by *Pseudomonas aeruginosa*, *Morganella morganii*, and *Bacteroides* sp. Also, the comparison of the metaproteome to in vitro cultures of *P*. *aeruginosa* and *M*. *morganii* revealed that iron limitation is one of the major challenges for the bacteria in the bladder environment, which they overcome by high expression of genes for siderophore production and the respective receptors. Apart from that, each bacterium employed a different strategy for nutrient acquisition, while, for example, *P*. *aeruginosa* abundantly expressed proteases and amino acid uptake transporters, *M*. *morganii* is able to take up sugars and degrade urea. Moreover, the comparison of urine protein profiles of long-term catheterized patients and healthy individuals revealed elevated level of proteins that might be associated with the innate immune system. In another study, Patnode and colleagues [10] recently combined metaproteomics with multispecies transposon mutagenesis to identify which bioactive carbohydrates in 34 dietary fiber preparations were degraded by specific *Bacteroides* species in the colon, with the ultimate goal of developing microbiota-directed foods. The authors examined how gene expression and the metaproteome changed in a defined microbial community when the mouse host was feeding on different dietary fiber preparations, showing, for example, that arabinan from pea fiber is a key nutrient source for at least 3 *Bacteroides* species, and how these 3 species directly compete with each other for polysaccharides in the gut.

In addition to microbial gene expression, metaproteomics can also measure host gene expression helping to infer host–microbiome interactions that underlie disease. Having both host and microbial gene expression data enables researchers to test for correlations between gene expression in the host and microbiome. Recently, Pathak and colleagues [11] used metaproteomics to identify the gene expression responses of pathogens and the host during ventilator-associated pneumonia (VAP). They identified 66 unique pathogen peptides using bronchoalveolar lavage (BAL) and endotracheal aspirate (ETA) specimens. In addition, they identified more than 3,000 human proteins in ETA, many of them associated with innate and adaptive immunity. These findings may guide future research in VAP diagnosis and antibiotic treatment alignment with specific pathogens. Using a similar metaproteomic approach, researchers proposed a set of bacterial and human proteins of the oral biofilm that allows the differentiation between healthy and caries-bearing individuals. The same authors also proposed that these findings may help in the development of personalized medicine in the prevention of tooth decay [12].

Other studies investigated the functional interactions between microbes and host associated with type 1 diabetes pathogenesis development [13,14]. Using a metaproteomics approach, Gavin and colleagues [13] identified clear signatures for new-onset type 1 diabetes in stool that can be useful in the development of therapies and diagnosis. Similarly, Tanca and colleagues [14] found that Clostridial butyrate biosynthesis enzymes were significantly reduced in diabetic mice as compared to nondiabetic mice [11], adding to the evidence that the intestinal microbiota is involved in the pathogenesis of type 1 diabetes and that reduction of butyrate synthesis may play a role.

As proteins are the main players of reactions and cellular processes, the identification of their subcellular localization is important for understanding their function. For example, Zhang and colleagues found that particular proteins in extracellular vesicles (EVs) from the intestinal mucosal–luminal interface were much more abundant in pediatric inflammatory bowel disease (IBD) [15]. Moreover, they were able to discern that it was the host proteins that were more abundant in EVs, whereas microbial proteins were less abundant. This study highlights the power of using subcellular compartment-resolved metaproteomics to reveal associations/interactions between the microbiome and the host. Similarly, as pathogens can induce alterations in specific subcellular compartments of their hosts, the use of subcellular fractionation can help us to better understand these interactions.

Metaproteomics can also provide information on microbiome composition by quantifying biomass contributions of individual species. Since protein comprises the majority of cellular material in most microorganisms, proteins identified and quantified with metaproteomics can be used to estimate biomass of species if data are correctly analyzed [16]. This approach provides information that is inherently different than the approximations of cell or genome copy counts that are provided by commonly used sequence-based methods.

Metaproteomics can also be used to infer natural carbon isotope composition of peptides and proteins and thus of specific species. Recently developed approaches make use of this to (1) link microbial species in communities to the environmental carbon sources that they consume by protein stable isotope fingerprinting (Protein-SIF) [17]; and (2) to follow incorporation and interspecies transfer of isotopically labeled substrates in microbial communities by protein-based stable isotope probing (Protein-SIP) [18]. Protein-SIP was recently used to detect differential incorporation of ^15^N labeled dietary protein in members of the mouse microbiota [19].

## What are the prerequisites for starting a metaproteomics study?

For a successful metaproteomic experiment, the experimental design must consider the availability of necessary instrumentation and a dedicated protein sequence database needed for protein identification.

For instrumentation, it is critical to have access to both a high-resolution liquid chromatography (LC) system and high-resolution mass spectrometry (MS). Although there are several experimental LC-MS/MS approaches for metaproteomics, 1 particularly powerful approach is 1D-LC with long analytical columns and stationary phases with small particle size. Long columns allow separating the complex metaproteomic peptide mixtures with high resolution using long LC gradients [20]. To work with long columns, the nano-LC system must be able to sustain high back pressures, which can range from 200 to 1,000 bar depending on the flow and column used. If particularly complex samples (soil and intestinal microbiomes) are analyzed, it can be critical to use an LC system that enables two-dimensional separations of peptide mixtures [20]. The mass spectrometer must provide high resolution (>25,000), accuracy (<10 ppm), sensitivity, and fast scan speed (>10 Hz). Currently, the most suitable instrument types are hybrid Orbitrap mass spectrometers, certain Q-TOF instruments, and recently developed instruments that combine ion mobility with time of flight [21]. More recently, data independent acquisition (DIA) has been applied in metaproteomics studies [22,23] with the promise of increasing metaproteome coverage and improving protein quantification.

Proteins are usually identified by searching the acquired mass spectra against a protein sequence database. In this strategy, experimental spectra are compared to theoretical spectra predicted from a comprehensive protein database. The database should contain the expected protein sequences for a given sample. Research over the last few years has shown that, ideally, the protein sequence database used for metaproteomics should be derived from a metagenomics/metatranscriptomics sequencing experiment of the same samples used for metaproteomics [24,25]. The use of protein sequences from reference databases such as Uniprot has been shown to greatly reduce the number of proteins identified [26] and potentially increase rates of false positives and incorrect taxonomic assignment of identified proteins [27]. In special cases, for example, when the study is performed on gnotobiotic animals inoculated with a defined microbial community, a database assembled from reference databases can be used [10,28].

## What does a general metaproteomics workflow look like?

**Fig 1:**
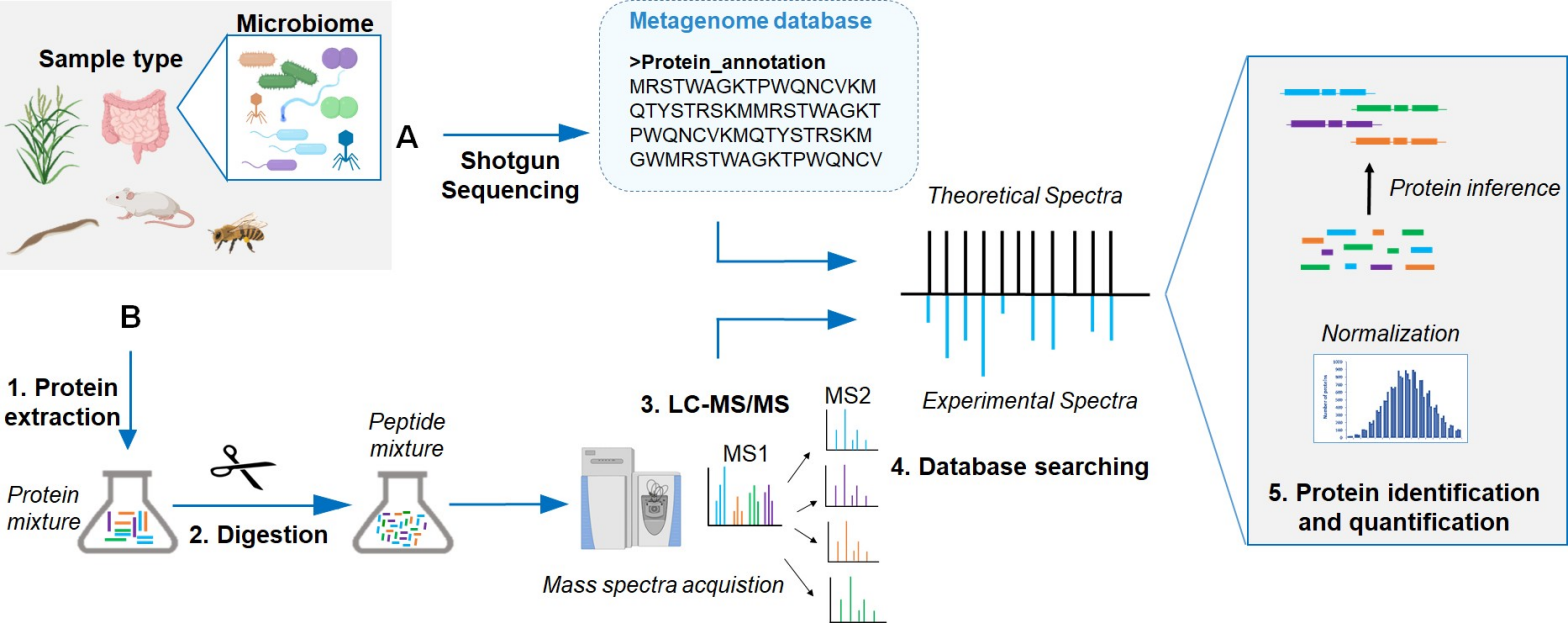
General workflow employed in metaproteomics experiments. (A) Metagenome shotgun sequencing can be used to generate the reference database for metaproteomics. (B) Metaproteomics workflow including generation of peptides and high-resolution MS analysis. LC, liquid chromatography; MS, mass spectrometry.

The most common metaproteomics workflow consists of sample collection and preservation, cellular lysis, protein extraction, tryptic digestion of proteins into peptides, peptide separation by LC, and analyses of peptide masses (MS) and their fragments (MS/MS) by mass spectrometry (Fig 1). The success of a metaproteomic study depends on 3 general factors: efficiency of protein extraction, efficiency of separation, and unambiguous identification [29].

The efficiency of protein extraction from tissues or environmental samples is dependent on sample preservation, available sample amount, and composition. Adequate sample preservation during collection is critical to avoid protein degradation during storage. Some preservatives have been tested to ensure sample integrity without freezing when necessary [30], although flash freezing of samples remains a preferred preservation method. Similar to other meta-omics approaches such as metagenomics, much progress has been made in reducing the input amounts needed for sample preparation. Current filter-aided or cartridge-based sample preparation protocols can work with just a few milligrams of sample (e.g., tissue or stool), while also efficiently removing interfering compounds. Some cell types are more easily disrupted, such as animal cells or gram-negative bacteria, whereas others, such as fungi, plant cells, or gram-positive bacteria, require harsher treatments for cell lysis. To reduce bias against specific cell types during extraction, it is critical to optimize protein extraction protocols for specific samples. For example, cell lysis by ultra-sonication in sodium dodecyl sulfate (SDS) lysis buffer has been shown to work well for metaproteome analyses of intestinal communities [31].

To achieve a high number of peptides, and consequently, a high number of proteins identified, sample complexity needs to be reduced by separating either proteins or peptides. The most common separation approach consists of on-line separation of peptides by nano-LC using a reversed-phase column (RP) and injection into the mass spectrometer. In addition, a second on-line separation step (2D-LC) can be used by adding a second column (e.g., strong cation exchange) upstream of the RP column [32]. Before on-line separations, proteins are usually prepared and digested following the filter-aided sample preparation protocol (FASP) [33]. As an additional step, proteins can be pre-separated by 1D-SDS PAGE gel electrophoresis and peptides prepared by in gel digestion prior to nano-LC (GeLC method). The separation approach of choice will depend on the sample and LC instrumentation available [20].

Each metaproteomics run will generate tens to hundreds of thousands of mass spectra of peptides and their fragments that are then used for peptide and protein identification. For identification, mass spectra are computationally matched to theoretical mass spectra derived from a protein sequence database. Development of efficient search algorithms is a very active research field providing a great diversity of commercial and open-access software. As discussed in question 2, the choice of nano-LC system, MS instrumentation, and reference database will determine the power of protein identification and the ability to discriminate homologous proteins from different organisms.

## How accessible is metaproteomics to the general scientific community, and how much does it cost as compared to other meta-omics technologies?

Similar to DNA or RNA sequencing, MS-based research is often facilitated by dedicated research service core facilities available at many institutions. Thus, to conduct a metaproteomics experiment, the researcher does not necessarily need a mass spectrometer in their laboratory. Mass spectrometry research centers can provide the analyses as a service; however, many centers currently do not have adequate experience with the preparation, acquisition, and analyses of samples and data for metaproteomics, and thus, it will be up to the researcher to guide the process through frequent communication with facility staff. A frequent mistake is to transfer approaches developed for proteomics of individual organisms or tissues directly to metaproteomics samples, which have additional challenges such as sample matrix, diversity of cell types in the sample, and protein inference issues caused by the presence of large numbers of homologous proteins in the sample and protein sequence database.

The costs for metaproteomics analyses per sample are similar to those of metagenomics or metatranscriptomics experiments, and rapid developments in the area of LC and MS are decreasing overall measurement costs. One of the major cost drivers in metaproteomics is the amount of run time needed on the LC-MS/MS system per sample. The amount of run time needed is changing in recent years, for example, runs in the past would often take 24 hours or more per sample for protein identification, whereas today runs of 2 to 6 hours are often sufficient. The hands-on time for metaproteomic sample preparation is around 1.5 to 2 days, and large numbers of samples can be prepared in parallel [34]; thus, personnel costs associated with sample preparation are also similar to other meta-omics approaches.

## What do the data look like, and how can they be analyzed?

Many proteomics software packages, such as MaxQuant [35] and Proteome Discoverer (Thermo Fisher Scientific, Bremen, Germany), allow qualitative and quantitative exploration of metaproteomic data. The resulting tables can be exported and further analyzed with other software, including general statistical software such as R [36] and specialized gene expression analysis software such as Perseus [37]. Open-source software dedicated to metaproteomics, such as MetaProteomeAnalyzer [38] and MetaQuantome [39], provide tools for data analyses and interpretation. For a review of specialized metaproteomics software, see [40].

**Fig 2:**
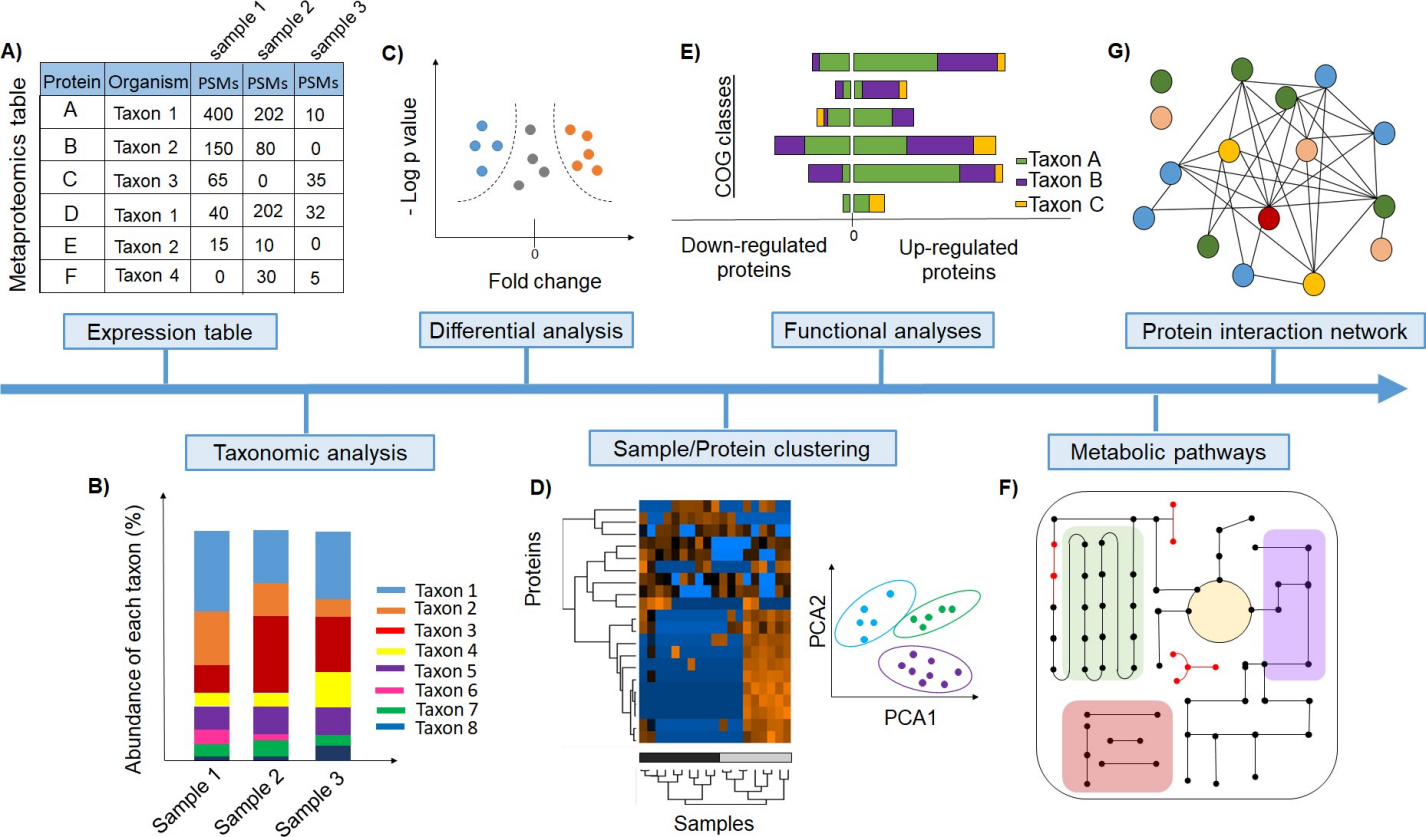
Examples of different analysis approaches to extract and help the interpretation of biological information from metaproteome datasets. (A) Table containing all proteins identified per taxon and associated PSMs. (B) Microbiome composition in terms of biomass contributions can be provided by the summed relative protein abundance of each taxon. (C) Volcano plots display abundance differences and thresholds derived from statistical tests corrected for multiple hypothesis testing and allow for the identification of differentially abundant proteins between treatments/conditions. (D) Multivariate analyses, visualized by PCA plots and hierarchical clustering, help to classify samples according to protein abundance differences. (E) COG represent functional protein groups across different microbes. (F) Pathway reconstruction supported by pathway databases. (G) Analyses of protein–protein interaction networks by mapping protein functional categories. These types of tests and visualizations are available through R packages [30] or the free GUI-based software Perseus [31] customized for proteomic data analysis. COG, Clusters of Ortholog Groups; PCA, principal component analysis; PSM, peptide-spectrum match.

After database searching, the output usually consists of a large table that provides, for each protein, an identity, an annotation, and the number of peptide-spectrum matches (PSMs), among other features (Fig 2A). Quantitative metaproteomics experiments interrogate the whole metaproteome and identify which proteins show differential abundances between different conditions. For differential metaproteomics, spectral counting approaches seem to be more robust for estimating abundances compared to peptide intensity approaches [16]; however, this has to be tested more extensively. Spectral counting approaches use the number of PSMs mapped to each protein as the quantitative value, which is usually normalized to protein length and total PSM number in the samples [41]. In metaproteomics, the normalized spectral abundance factor (NSAF) is frequently used [42]. Metaproteomic datasets, like most count-based microbiome datasets, are compositional, and thus, appropriate statistical methods should be used to address data compositionality issues [43].

The richness of information provided by the metaproteomic data allow researchers to look at complex biological questions that can be addressed using protein abundances (Fig 2). For example, species abundances can be calculated by summing the relative protein abundances for each species; these estimates can then provide the microbial community composition in terms of biomass contributions of different taxa (Fig 2B). For differential abundance analyses of proteins, various statistical methods with correction for multiple hypothesis testing can be used to identify proteins that differ significantly in abundance between treatments, conditions, and body locations. Abundance differences and significance thresholds can, for example, be displayed using a volcano plot (Fig 2C). Differential abundance analyses are often a critical tool for identifying genes/proteins of particular relevance under a given condition and thus helps to narrow the focus. Understanding how samples/treatments as a whole differ can also help narrow the results. For this, multivariate analyses visualized by principal component analysis (PCA) plots and hierarchical clustering help characterize the differences across samples (Fig 2D). For example, hierarchical clustering identifies similarities and differences among all samples by separating them from different experimental states based on their protein abundance values. This enables the identification of protein clusters of similar abundance changes across treatments. These types of tests and visualizations are accessible through R packages or the free GUI-based software Perseus [37] customized for proteomic data analysis.

For functional analyses, proteins can be classified into Clusters of Ortholog Groups (COG). Each COG represents a group of orthologous proteins from different microbes sharing the same functional characteristics (Fig 2E) [44]. Similarly, Gene Ontology [45] and eggNOG [46] provide functional annotations. Furthermore, analyses of specific metabolic pathways through manual reconstruction or use of automated tools, such as Pathway Tools [47], can provide a more in-depth visualization of the functional state of the metaproteome. The commonly used pathway databases used to support pathway reconstruction are MetaCyc [48] and KEGG pathways [49] (Fig 2F). Construction of protein–protein interaction networks can give insights about protein function in biological processes. Protein–protein interactions can be visualized by mapping, for example, COG categories against the String database [50]. In addition, a new tool called MicrobioLink [51] offers a pipeline for downstream analyses of host–microbiome functional interactions. Information on additional tools for functional analyses and their validation can be found in a recent comparative study by Sajulga and colleagues [52].

Lastly, the integration of metaproteomics with other “meta-omics” approaches, such as metagenomics and metatranscriptomics, are growing in popularity since it allows the investigation of complex mechanisms across different molecular layers. Several workflow analyses for the integration of meta-omics datasets have been proposed [53–55].

In summary, metaproteomics is a rapidly growing field that allows to characterize microbial communities and host-associated microbiomes on multiple levels. The enabling technologies (LC and MS/MS) see major improvements every year, while also the number of metaproteomics experts is growing, which will make metaproteomics measurements more broadly accessible on the near term. The nascent metaproteomics community has started to organize, and a first set of inter-lab comparison studies is under way to test and validate differing metaproteomics workflows, with the ultimate goal to consolidate and standardize some of the approaches. At the same time, new metaproteomics wet lab and computational methods are continuously being developed to provide additional capabilities. We are confident that metaproteomics will continue to grow in its importance as a tool for the study of host-associated microorganisms.
